# Research capacity for sexual and reproductive health and rights

**DOI:** 10.2471/BLT.15.163261

**Published:** 2016-06-02

**Authors:** Rita Kabra, Moazzam Ali, A Metin Gulmezoglu, Lale Say

**Affiliations:** a Department of Reproductive Health and Research, World Health Organization, avenue Appia 20, 1211 Geneva 27, Switzerland.

Research is important for improving health outcomes and is a critical element of a functioning health system. Without locally generated data and analysis, well-intentioned programmes do not often respond to the realities where they are implemented.^1^ Hence strengthening research capacity in low-and middle-income countries is one of the most powerful, cost–effective and sustainable measures of advancing health, health care and development.^2^

*The world health report 2013: research for universal health coverage* referred to research capacity as “the abilities of individuals, institutions and networks, nationally and internationally, to undertake and disseminate research findings of the highest quality”.^3^ The report provides examples of efforts that build research capacity by national and international agencies focusing on the particular element of capacity building. However, best results in capacity building are obtained when there are interactions between individuals, institutions and networks to support research. For example, graduate and postgraduate training are more likely to be effective when the host institutions are also strong.^3^

Since 1990 the number of initiatives on strengthening research capacity in low- and middle-income countries has increased to over 300.^4^ However, in many countries there is still insufficient capacity to engage in research that will influence evidence-based policies and programming at country level.^5^ Lack of funding, expertise in preparing manuscripts for publication^6^ and protected time for research pursuits, as well as the infrastructure of institutions, are key constraints faced by researchers.^7^

Traditionally, investments and efforts to strengthen research capacity in these countries have mostly been focused on individual research training such as masters, doctorate and fellowship programmes.^8^ Lately the Wellcome Trust African Institutions Initiative^9^ has emphasized institutional development as an important component of strengthening capacity.

Although there is an increase in initiatives to strengthen health research capacity, such initiatives are scarce in the field of sexual and reproductive health and rights. Mapping 303 global initiatives – which aims to strengthen health research in low-and middle-income countries – showed that 35% of initiatives focused on infectious disease, while only 3% of the initiatives specifically focused on reproductive, maternal and child health.^4^

To improve reproductive health by supporting national and/or regional research, the human reproduction programme (HRP) was formed in 1972 as the United Nations Development Programme/United Nations Population Fund/United Nations Children’s Fund/World Health Organization (WHO)/World Bank Special Programme of Research, Development and Research Training in Human Reproduction. The programme is the main instrument within the United Nations system for research in human reproduction and has a global mandate to lead research and conduct research capacity strengthening in the field of sexual and reproductive health and rights.^10^^,^^11^ The programme consists of an alliance comprising: (i) institutions receiving support from HRP under its research capacity strengthening schemes; (ii) institutions and individuals who have engaged strongly with HRP on various multi-country thematic research projects; (iii) official WHO collaborating centres working with the WHO Department of Reproductive Health and Research; and (iv) WHO Regional and Country Offices.^10^

Sexual and reproductive health research in low- and middle-income countries faces several key challenges in achieving high-quality research and publications. Often, the research is underfunded, emerging institutions lack visibility and networking opportunities, there are few training opportunities for individuals and grant termination leads to inability to conduct or continue independent research. This paper outlines how HRP addressed these challenges by consolidating and modifying its research capacity strengthening approach in 2015.

The renewed approach is focusing on a sustainable approach towards institutional development with an emphasis on regional networking through the HRP Alliance.^10^ The approach employs a flexible and context dependent process to address priority needs and build national self-reliance in research on all aspects of human reproduction, as well as the readiness of an institution to conduct research.

The approach is comprehensive compared with other global research capacity strengthening initiatives. HRP uses a four pronged approach for capacity building that: (i) strengthens institutional infrastructure; (ii) develops and enhances skills of researchers for conducting quality research; (iii) ensures sustainability through long-term grants; and (iv) promotes networking among the research community by linking the institutions.

HRP considers strong institutions to be not only a prerequisite for research capacity strengthening but a necessity to retain trained staff, because individuals do not work alone and their skills and capacity are influenced by the institution and systems in which they operate. HRP offers a variety of research capacity strengthening grants. Institutional support is provided via long-term institutional development grants.^12^ The grant provides the recipient institutions a stable source of support for a period of up to 10 years. The grant seeks to strengthen the institution’s capacity across all resources needed for high-quality research such as infrastructure, human skills, financial support, information technology, equipment such as computers and libraries’ access to scientific literature.

At the outset of the long-term institutional development grant, the institution identifies its goals and priorities for strengthening research capacity in sexual and reproductive health. The grant supports training workshops on good research practices, proposal development, data analysis and management and ethics in research for sexual and reproductive health. Based on the institution needs, special courses on systematic reviews, research methods, scientific writing and sensitive issues such as gender-based violence are provided. HRP encourages and supports grantees to design and undertake research, and to write and publish research findings in national and international journals.

As institutions gain sufficient experience and capacity, they are invited to participate in global multi-country studies conducted by HRP. For example, in 2014, *Cellule de Recherché en Santé de la Reproduction* – an institute based in Conakry, Guinea – was included as a study site for the WHO multi-country study on disrespect and abuse during childbirth. Together with other sites, the institute participated in developing the research protocol. This approach helped the national researchers to gain confidence and hands on experience in cross-country research.

HRP encourages institutions that have strengthened their research capacity (so-called mature centres) to become centres of excellence supporting the region and serve as a WHO collaborating centre for sexual and reproductive health and rights. One example is *Institut de Recherche en Sciences de la Santé* in Ouagadougou, Burkina Faso. With the support of a long-term institutional development grant, the institute’s research capacity has been strengthened, leading to participation in WHO-led multi-country research on postpartum family planning. The institute is now offering a master’s degree programme in public health open to international students and is collaborating actively with neighbouring countries in regional research. In 2015, WHO invited the institution to apply for official WHO collaborating centre status.

To create a critical mass of researchers conducting relevant national policy research, masters and doctorate programmes are increasingly supported via research training grants given to students. After completed studies, re-entry grants help trainees to establish themselves as researchers in their country of origin, thus preventing migration of trained researchers. If and when an institute needs external support, it is linked with a mature centre via a research project mentoring grant. The location of the mature centre is preferably in the same region, facilitating regional network and partnership formations and promoting south–south cooperation. For example, in Paraguay, the Center for Population Studies (CEPEP) – a long-term institutional development grantee, was supported by *Centro de Estudios de Población* in Argentina from 2009–2014, through a research project mentoring grant. This grant enabled CEPEP researchers to acquire the technical skills needed to conduct high-quality scientific research in sexual and reproductive health.

Emerging institutions can connect with mature centres and the wider research community via the HRP Alliance platform. The platform increases the emerging institutions’ visibility and engagement, decreases researchers’ isolation and promotes knowledge-sharing. For example, an institution from Côte d’Ivoire is receiving mentorship and support from the institution in Burkina Faso through the HRP Alliance.

To promote partnerships and networking at regional level, competitive intraregional grants are provided (sometimes coupled with the mentoring programme). In 2014, for example, HRP announced a call for proposals on “Strategies to improve quality of care in sexual and reproductive health and rights”. 

The efforts by HRP to strengthen institutional and individual research capacity in reproductive health have resulted in mature and sustainable research institutions. These institutions participate in global health research and multicentre studies. They have been able to influence national and international research agendas and policies. For example, centres in Argentina and Guatemala have been instrumental in the implementation of multicentre studies on caesarean section and a trial coordinated by Oxford University, United Kingdom of Great Britain and Northern Ireland, which evaluates the use of magnesium sulfate for the treatment of pre-eclampsia. Centres in China have conducted numerous studies on contraception. Some countries are still developing their research capacities, particularly those that have been affected by conflicts, war or natural disasters. In such countries, developing research capacity will take longer than in other countries.

HRP will continue to support institutions in low-and middle-income countries and train researchers so that they can conduct high-quality research and disseminate research evidence supporting national and international sexual and reproductive health policy and programme development. The HRP Alliance will help research to contribute to the broader context of improving the health of women, children and adolescents.
